# Work schedule flexibility and teleworking were not good together during COVID-19 when testing their effects on work overload and mental health

**DOI:** 10.3389/fpsyg.2022.998977

**Published:** 2022-10-28

**Authors:** Jesús Yeves, Mariana Bargsted, Cristian Torres-Ochoa

**Affiliations:** ^1^Faculty of Psychology, Diego Portales University, Santiago, Chile; ^2^Millennium Nucleus on the Evolution of Work, Santiago, Chile; ^3^Psychology School, Adolfo Ibáñez University, Santiago, Chile

**Keywords:** work schedule flexibility, teleworking, work overload, mental health, COVID-19, new ways of working, longitudinal study, working from home

## Abstract

The COVID-19 pandemic has driven organizations to implement various flexible work arrangements. Due to a lack of longitudinal studies, there is currently no consensus in specialized literature regarding the consequences of flexible work arrangements on employee mental health, as well any long term potential impacts. Using the Job Demand-Resource Model, this study documents consequences of the implementation of two types of flexible work arrangement: work schedule flexibility and teleworking on employee mental health over time, and the mediating role played by work overload during the accelerated implementation of flexible work arrangements in the course of the COVID-19 pandemic. Using a longitudinal design and probabilistic sampling, 209 workers participated in this study, twice answering a flexible work arrangement and mental health questionnaire during the pandemic. Findings of this moderated-mediation suggest that work schedule flexibility generates positive effects on mental health over time due to decreased work overload, but only for employees not working from home. These results offer theoretical and practical implications applicable to organizations considering implementation of flexible work arrangements, particularly with regard to how these flexible practices could support a balance between demand and resources, their impact on work overload, and employee mental health over time.

## Introduction

There is no doubt the COVID-19 pandemic has had a global impact on work (International Labour Organization, 2020a; Eurofound, 2021). Flexible work arrangements (FWAs) have been crucial to enable employers to rapidly adapt to the enormous unexpected changes (Society for Industrial and Organizational Psychology, 2021). FWAs are a form of work flexibility without rigid boundaries around workspaces and work schedules (Kotera and Correa Vione, 2020). Existing literature on FWAs has focused primarily on two different types of flexibility: spatial flexibility, in terms of “where” to work, allowing employees to work outside the physical organizational boundaries; and temporal flexibility, or “when” work is performed, allowing employees to manage their schedule (Rau and Hyland, 2002; Van Steenbergen et al., 2018; Kotera and Correa Vione, 2020). Teleworking is an example of spatial flexibility, permitting employees to fulfill their roles outside the physical organizational boundaries, such as working from home (WFH). Work schedule flexibility (WSF) is an example of temporal flexibility, allowing employees to control their working hours (Shifrin and Michel, 2021).

Systematic reviews of WFH and WSF show both positive and negative consequences for employees (Kotera and Correa Vione, 2020; Oakman et al., 2020; Lunde et al., 2022; Shiri et al., 2022). In particular, evidence suggests there are positive impacts of such arrangements when they are implemented under the right conditions. According to Kossek and Michel (2011), the keys to successful implementation of FWAs are: voluntarity, formalization, mutual agreement between employer and employee, and the presence of an organizational culture supporting flexibility. Kossek and Michel (2011) further found positive outcomes are facilitated by employer willingness to adjust job design, fitting the job to the particular needs of the employee.

Prior to the COVID-19 pandemic, Demerouti et al. (2014) proposed that FWAs could negatively impact both job demands and job resources. FWAs imply time pressure, unanticipated tasks, interruptions, task switching, diverse roles, isolation and reduced face-to-face interaction. In such circumstances, FWAs would reduce job resources such as overall communication between the organization and its workers and social support typically present in an in-office job and would increase job demands such as work overload (Demerouti et al., 2014).

However, as a consequence of the pandemic, some FWAs, such as teleworking, were not optional, due to the need to remain in our homes for safety reasons, a condition known in the literature as “Mandatory Work From Home” or “Working From Home (WFH).” Because WFH is not identical to teleworking, transferability of data from studies conducted prior to the pandemic is now complicated (Kniffin et al., 2021). As Michinov et al. (2022) stated, WFH differs from teleworking in that it is mandatory, and requires fulfilling multiple personal and work roles simultaneously, with restricted mobility and physical space.

Pulido-Martos et al. (2021) also hold that the abrupt and unplanned adoption of the WFH modality transformed the psychosocial environment at work, shifting various labor and personal resources in workers, making it essential to explore its consequences on employee health. Under normal conditions, correct implementation of teleworking requires precise planning, socialization, piloting, and evaluation (Greer and Payne, 2014). Therefore, as per Demerouti et al. (2014), it is plausible to postulate that, during the pandemic, FWAs such as WFH may have given rise to increased stressors on employees through work overload, negatively impacting worker well-being.

Pre-pandemic literature about FWAs and their impact on employee health has shortcomings and is scant. There are a few studies addressing the different types of FWAs (Van Steenbergen et al., 2018; Shifrin and Michel, 2021), but most have been cross-sectional (Van Steenbergen et al., 2018; Shiri et al., 2022), which does not allow exploration of employee impacts over time, or establishment of causal inferences from the findings (Taris et al., 2010; Hayes, 2013). In addition, pre-pandemic literature focuses mainly on the association between FWAs and mental health rather than seeking explanatory models to clarify these relations between the two (Beckel and Fisher, 2022). This permits only a limited understanding of what interventions could improve this relationship.

The identified gap in scholarly literature, and the rapid implementation of FWAs during the pandemic make it advantageous to conduct research on the impact of FWA work modalities on employee mental health in a quest for improved outcomes. The primary aim of this study is therefore to identify the impact of FWAs on mental health over time, especially WSF and WFH, insofar as they represent the “when” and “where” to work of FWAs, respectively (Rau and Hyland, 2002; Van Steenbergen et al., 2018; Kotera and Correa Vione, 2020). A second aim is to explore the potential role of work overload as a mediating variable between these FWAs and mental health. In particular, the study investigates this relationship using a moderated mediation model where WSF is a possible predictor of positive mental health. This will be accomplished through a two-waves longitudinal study (commencing with the pandemic and 6 months later with work overload as mediating variable in this relationship). In addition, the study examines whether WFH is a moderating variable in the hypothesized relationship between WSF and work overload, such that the relationship between WFS and mental health *via* work overload is weaker for those working from home.

The contributions of this research are several. First, the study adds to the body of knowledge of FWA literature using a longitudinal design, granting a better understanding of the causality between two different FWAs and mental health. This represents an important contribution to the field of FWAs, as, according to recent systematic reviews, studies of the impact of FWAs on mental health have mostly relied on cross-sectional designs (Oakman et al., 2020; Lunde et al., 2022; Shiri et al., 2022). Second, although Kossek and Michel (2011) indicated that combining various types of FWAs can maximize positive outcomes for both employees and employers, a recent meta-analysis by Shifrin and Michel (2021) found there is not yet enough evidence to support this claim. The implementation of FWAs involves a change in job demands, prompting a need for study (Demerouti et al., 2014; Van Steenbergen et al., 2018). Therefore, the results of this study may contribute to the understanding of whether the combination of two different FWAs (such as WSF and WFH) during the pandemic reduces or increases a job demand such as work overload. The third contribution of this study lies in the proposal of a first stage moderation-mediation model (Hayes, 2013) elucidating how WSF and WFH could affect mental health over time through work overload.

### FWAs from a job demand-resource model’s perspective

The theoretical framework for this research is the Job Demand-Resource Model (JD-R), which Demerouti et al. presented in a 2001 study indicating that work stress is due to a mismatch between job demands and job and personal resources. According to this model, the concept of job demands refers to the physical, psychological, organizational, or social aspects of work that require sustained effort and entail physiological and psychological costs. Resources are defined as the physical, psychological, organizational, or social aspects of work that can (a) reduce job demands and associated physiological and psychological costs, (b) be instrumental in achieving job goals, or (c) stimulate personal growth, learning, and development (Bakker and Demerouti, 2007, 2013; Bakker, 2011).

In 2014, Demerouti et al. conducted an analysis of how job demands and job resources shifted under the new ways of working, finding that FWAs impact working conditions, work-family balance, and well-being using the JD-R model. They found that FWAs engendered several significant changes in job resources such as autonomy, media technology, and social support. In addition, the study also found that FWAs altered job demands, such as increases in pressure to respond quickly, unanticipated tasks, interruptions, task switching, diverse role demands, and lack of control over communication. To study the causal relationships between the consequences of the implementation of FWAs and employee well-being, Demerouti et al. (2014) recommended longitudinal design studies.

FWAs are strategies facilitating work, and, if applied correctly, function as resources. These enhance work engagement and well-being, reduce work overload, and improving worker well-being and performance (Michinov et al., 2022). However, the literature shows that both WSF and teleworking could have either positive or negative outcomes (Shiri et al., 2022). As several studies indicate, new resources such as FWAs could affect demands such as work overload. For example, Hayman (2010) reported a potential decrease in work overload when WSF is implemented. In the case of teleworking the literature has shown mixed results, whereas Mann et al. (2000) and Tavares (2017) stated that teleworking can increase work overload, while Gajendran and Harrison (2007) found opposite evidence. The current changes in the way work is performed (Van Steenbergen et al., 2018), makes pertinent a study of how these two types of FWAs interact in their effects on work overload. Gaining further evidence of the role of FWAs as an either a demand or resource and concomitant potential impacts on mental health from work overload is therefore apposite.

### WSF, work overload, and mental health

FWAs are generally defined in terms of “when” and/or “where” work is completed. In particular, “when” to work has been referred to by literature as flextime or WSF, a concept referring to employee ability to tailor their work schedule to their own needs to varying extents (Krausz et al., 2000; Rau and Hyland, 2002; Van Steenbergen et al., 2018). Compressed work week, a flexible start and end to the workday, and a variety of times to take breaks are among the most common WSF practices (Jung Jang et al., 2012).

WSF can be implemented according to formal or informal organizational practices (Grzywacz et al., 2008). Joyce et al. (2010) stated the types of WSF can be differentiated based on the level of control the employee has over their own schedule. The ability to change it with higher autonomy is known as self-scheduling. A variable work schedule with a core period in which all employees must be available, and a plan schedule requested in advance by the organization is known as flextime (Joyce et al., 2010).

Katz and Kahn (1978) established that work overload occurs when too much is expected of a worker within the available time, or when a job demand exceeds employee capability. Work overload has been referred to as a traditional job demand since it is characterized by the need to work faster, provide more agile responses, perform multiple tasks and/or carry out several projects at the same time (Ingusci et al., 2021). In the JD-R model, work overload is categorized as one of most relevant demands for most people’s work environment since it involves considerable physical and/or psychological effort to address (Bakker et al., 2003, 2005; Bakker and Demerouti, 2007, 2017). Similarly, some studies have identified work overload as a potential stress factor (Osca et al., 2003; Osca, 2012).

Although there are few studies exploring the potential effect of WSF on work overload, using the JD-R model makes it possible to propose a negative relationship between these two variables based on the increase in the autonomy and control resources that WSF implies for workers (Demerouti et al., 2014). Therefore, time autonomy can be classified as a crucial labor resource to cope with job stress (Bakker et al., 2005) and WSF can be classified as a job resource that buffers demands such as high work pressure or work peaks. Considering these theoretical frameworks and the results of previous studies, we propose the following hypothesis:

*H1*: WSF will be negatively related to work overload.

The concept of mental health will be referred to here as a state of well-being in which the individual can fulfill his or her own abilities, cope with the normal stresses of life, work productively and fruitfully, and contribute to his or her community (World Health Organization, 2004). According to the JD-R model, the workplace imbalance occurring when job demands exceed job resources is the main predictor of mental health impairment such as stress and burnout, since job demands involve sustained psychological strain (Demerouti et al., 2001; Bakker and Demerouti, 2007; Bakker and Demerouti, 2013). Similarly, when work overload, considered as one of most relevant demands for most people’s work environment (Bakker et al., 2005), exceeds job resources, the result is a gradual health impairment process with negative outcomes connected to psychological ill-health symptoms such as behavioral stress, and burnout (Bakker et al., 2014; Bakker and Demerouti, 2017).

These postulates have been tested in a meta-analysis by Bowling et al. (2015), who found that work overload was related to psychological and physical health outcomes. Several studies have shown similar results: Melchior et al. (2007) showed how psychological job demands contribute to an increased risk of two common psychiatric disorders such as major depression and generalized anxiety disorder; Shultz et al. (2010) documented that higher work overload is linked to sleep problems, irritability, anxiety, and personal problems; Su et al. (2018) found a positive relationship between work overload and burnout and a negative one with mental health; and Alarcon (2011) established that job demands play a crucial role in the prediction of burnout, work overload being one of the most important predictors of burnout. Considering the postulates of JD-R model and the previous empirical evidence, we proposes a second hypothesis:

*H2*: Work overload will be negatively related to mental health.

One of the positive aspects of WSF is the perception of employee control. According to Kossek and Michel (2011), this form of flexibility gives workers control over when and where to work, which is also related to the perception of autonomy. WSF has been linked to time autonomy, which allows workers to effectively manage their own time, coping with high job demands, such as work overload and daily stress for both work and leisure time. From this perspective, WSF has shown positive outcomes, such as lower absenteeism as employees can schedule personal appointments during normal business hours and make up the missed work time later in the day (Kossek and Michel, 2011). It has also been linked to better work-life balance and job satisfaction, and lower work overload and job-induced stress (Hayman, 2010), since it improves work recovery due to the control over the timing and content of breaks during the workday (Spreitzer et al., 2017).

However, several studies have reported a negative relationship between WSF and stress and mental health problems such as emotional stress (Jung Jang et al., 2012), work stress, and burnout (Grzywacz et al., 2008). Joyce et al. (2010) noted in their systematic review that self-scheduling and flexibility at the beginning and end of the workday had positive effects on physical and mental health, as these WSF increased the worker’s perception of control. Based on the proposed relations between WSF and work overload (H1), and work overload and mental health (H2), this positive effect of WSF on mental health could be mediated by work overload, as we proposed in Hypothesis 3:

*H3*: The relationship between WSF and mental health will be mediated by work overload.

### The role of WFH

Teleworking is defined as “working outside the employer’s premises making use of modern ICT” (Steidelmüller et al., 2020, p. 998), and represents the “where” of FWAs (Van Steenbergen et al., 2018). Although this mode of work can have many advantages, the nature of the work will dictate which jobs or professions will be better suited to teleworking than others. Highly suitable jobs or professions for telework include managerial and specialized professional information-based tasks, performed using devices such as computers and cell phones, which can be planned in advance and performed at any time of day and which require high levels of concentration and autonomy (Tavares, 2017).

Shiri et al. (2022) found FWAs may have varying impacts on work depending on its implementation. In the case of teleworking, Mann et al. (2000) found it involves an important change in communications; there is a reduction in face-to-face communications and communications can occur outside traditional times and spaces. As Eurofound (2020) stated, not all jobs transfer seamlessly to a teleworking model. Therefore, when implementing teleworking three main aspects of work should be adjusted: task contents of work (cognitive task, social interactions tasks, and physical tasks), methods of work, and tools required to perform a job. In terms of employee needs, teleworking requires training, ergonomic workstations, technological resources, and social support (Beckel and Fisher, 2022).

Pre-pandemic literature distinguishes advantages and disadvantages in the consequences of teleworking (Tavares, 2017; Steidelmüller et al., 2020). Among the former, there is evidence that teleworking can diminish commuting time, while increasing autonomy, productivity, and work-family balance (Steidelmüller et al., 2020). There is also a concomitant decrease in costs and distractions and an increase in the quality of the work environment and the perception of freedom (Mann et al., 2000).

Anderson et al. (2015) found that teleworking increased positive affect and diminished negative affect, and Tavares (2017) reported evidence of stress reduction, better work-life balance, better life control and job satisfaction in teleworkers. However, some disadvantages or risks regarding teleworking have been reported including increased demands on self-management, working longer and more intensively (Michinov et al., 2022). Other researchers noted a higher probability of working during illness (Steidelmüller et al., 2020), increased isolation, work overload, and a negative effect on career progression (Mann et al., 2000; Tavares, 2017).

Empirical findings on the advantages and disadvantages of teleworking are difficult to generalize in the context of mandatory work from home, especially those referring to its positive impacts (Kniffin et al., 2021). Recent studies have shown the perceived work overload of employees increased as a consequence of the greater demands they face when WFH, particularly during the pandemic (Wang et al., 2020; Sandoval-Reyes et al., 2021). As Demerouti et al. (2014) stated, electronic communication and employee availability anytime-anywhere, two main features of WFH, can extend the workday, and increase feelings of isolation, difficulties in setting structure, increasing work-, information-and social overload. In addition, the benefit of higher control over work is not achieved if WFH could transform flexibility into a way of channeling demands, rather than controlling them (Badawy and Schieman, 2021). Therefore, in the context of the JD-R model, WFH can be understood as a demand potentially increasing worker perceived stress, or dampening the relationship between labor and personal resources, as Pulido-Martos et al. (2021) found.

Schmitt et al. (2021) also found that WFH could increase cognitive overload and contribute to reduced worker productivity and well-being. Teleworking blurs the boundaries between work and home, and can lead to work overload, as it is associated with overtime (Kotera and Correa Vione, 2020; Wöhrmann and Ebner, 2021), lack of autonomy (Van Steenbergen et al., 2018), telepressure, and workplace monitoring (De’ et al., 2020), among others. Pursuant to JD-R model postulates and previous studies, we therefore propose the following hypothesis:

*H4*: The negative relationship between WSF and work overload will be moderated by WFH, so that this relationship will be stronger for those who are not WFH.

Finally, it is relevant to note that according to Joyce et al. (2010), FWAs are likely to have a positive effect on health outcomes provided as they increase work control and are implemented to support employees, rather than being directly focused on the interests of the organization. Nijp et al. (2012), in a systematic review reported that, taken together, work control time strategies do not have enough evidence about their impact on well-being, although when flextime is analyzed independently, there is moderately strong evidence for a positive association with health and well-being.

Literature demonstrating mixed results (Oakman et al., 2020; Lunde et al., 2022; Shiri et al., 2022), reveals the impact of WSF and WFH on mental health is complex. These results could be the consequence of models blending different types of FWAs (Nijp et al., 2012). It is therefore plausible to find the effects of some FWAs are related to the increasing or diminishing personal control or autonomy due to work overload. Hence, when two or more FWAs are present, they may potentiate positive effects, or reduce or nullify positive outcomes.

WFH during the pandemic was found to increase both quantitative job demands (work intensity and time pressure), and home demands (Abdel Hadi et al., 2021). It was also found to increase both cognitive demands and the use of technology for work (Dolce et al., 2020). While recovery after work increases health and well-being (Demerouti et al., 2014), it was particularly difficult to achieve this during the pandemic (Abdel Hadi et al., 2021). This work modality was found to be more demanding and stressful (Schmitt et al., 2021). Therefore, we propose that mandatory WFH can moderate H3, i.e., in the case of workers working from home, the effect of WSF on mental health *via* work overload will be different, specifically:

*H5*: The relationship between WSF and mental health through work overload will be moderated by WFH, so the indirect effect will be stronger when workers are not WFH.

**Figure 1:**
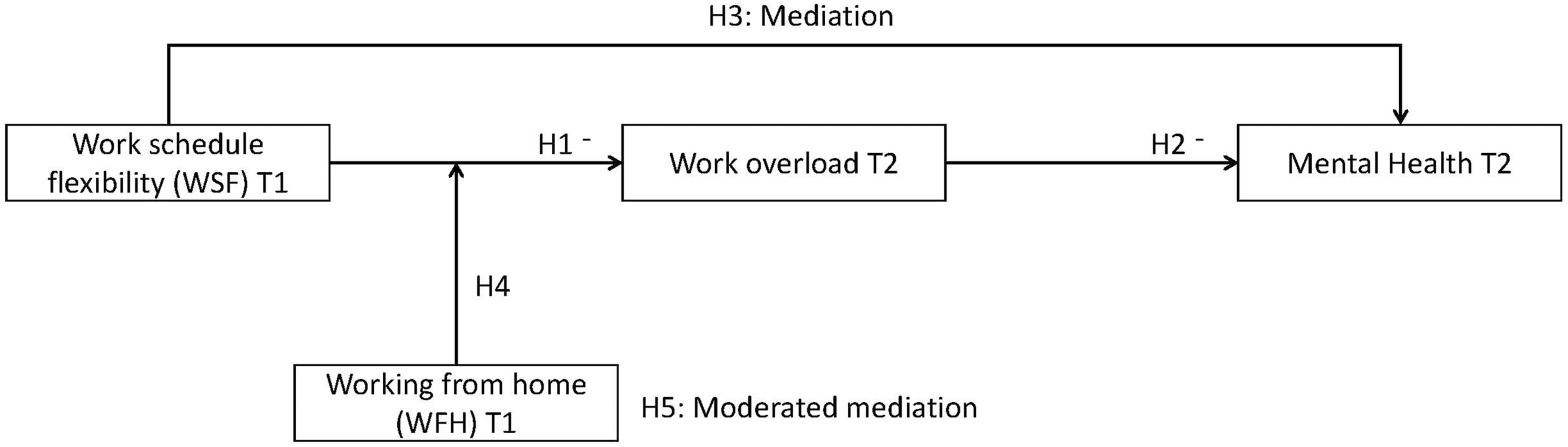
An illustration of the hypothesized relationships (H, hypothesis).

All hypotheses outlined above are visually represented in Figure 1.

## Materials and methods

### Participants and procedure

Data collection was carried out in November 2020 through a telephone survey applied to adults in all regions of Chile using the CATI (Computer-assisted Telephone Interviewing) system (Groves, 2014) by the Social Surveys Lab of Adolfo Ibáñez University. The sampling was strictly probabilistic by Random Digit Dialing (RDD), through the construction of a sampling frame of telephone numbers according to the market share of each company present in the country. Once contacted by telephone, subjects were invited to participate in the research after reading an informed consent form. In addition, they were informed that, should they decided to participate, they would receive another questionnaire in approximately 6 months, or in May of 2021. This six-month period was recommended by Demerouti et al. (2014) for analyzing consequences of implementation of FWAs and any causal relationship with employee well-being and has been used in previous studies related to antecedents and consequences of job stress (i.e., Lizano and Barak, 2012; Travis et al., 2016). The project was approved by the Research Ethics Committee at Adolfo Ibáñez University and anonymity and confidentiality of responses were guaranteed.

In Chile, the first case of COVID-19 in Chile was detected on March 3, 2020. Primary measures to contain the virus consisted of border closures, generalized confinement or quarantine, movement restrictions and implementation of the use of masks, the extent of which depended on the level of progress of the pandemic in the different localities of the country (Dirección De Presupuestos, 2022). In November 2020, in Chile (T1), 20.8% of formal workers had adopted WFH (Instituto Nacional de Estadísticas, 2021a), a figure that increased slightly to 21.1% by May 2021(T2; Instituto Nacional de Estadísticas, 2021b).

At Time 1, a total of 1,601 subjects responded to the survey. At Time 2, 25.4% or 406 of them responded again to the same survey. Attrition analyses (based on t-tests and chi-square tests) showed that there were no significant differences in terms of gender and age between workers who responded both times and those who responded only at Time 1. Respondents at Time 2 and non-respondents were also compared on the Time 1 model variables: work overload and mental health. None of the t-tests performed were statistically significant, meaning that there are no differences in the associations among the variables between those who dropped out and those who remained in the study.

Finally, to test the hypotheses, only those subjects who reported working on both occasions, excluding those who were self-employed, were selected, leaving an effective sample of 209 subjects. This effective sample was composed of 45.9% men, with a mean age of 42.9 years (SD = 13.28) at T1. 75.7% of participants worked in private companies, 22.4% in public organizations, and 2% in family businesses. Also, 20.6% of participants were professionals or scientists, 20.1% worked on services and commerce, 13.9% were administrative employees, 11.5% were technicians, 8.6% blue collar workers, 8.6% were non-qualified workers, 3.3% worked in agriculture and fishing; and 3.3% had managerial positions. Regarding the FWAs considered in this study, 45.9% had WSF at T1 and 47.8% at T2, from which 25.8% had WFH at T1 and 27.8% at T2.

### Measures

Work Schedule Flexibility. Incidence of WSF was measured with one ítem: “Regarding the labor measures that have been taken since the beginning of the pandemic to date, please indicate if work schedule flexibility has been applied in your case at present.” Answers are dichotomous, closed-ended (0 = No; 1 = Yes).

Working from home. WFH was measured with one item: “Regarding the labor measures that have been taken since the beginning of the pandemic to date, please indicate if working from home all week was applied in your case at present.” Answers are dichotomous, closed-ended (0 = No; 1 = Yes) to measure incidence of WFH during pandemic.

The application of dichotomous measures for these constructs, WSF and WFH before and during the pandemic, has been used in several prior studies for FWAs (e.g., Greer and Payne, 2014; Anderson et al., 2015; Solís, 2017; Wong et al., 2020; Pulido-Martos et al., 2021).

Work overload. Work overload was measured with 3 items pointing to work overload using Rizzo et al.’s (1970) scale, translated into Spanish by Peiró et al. (1986), using a 5-point Likert-type response scale (1 = Never – 5 = Always). An example of an item is: “I usually lack time to complete my work.” Cronbach’s alpha for T1 was 0.79 and for T2 was 0.80.

Mental health. Mental health was measured using the MHI-5 scale (Ware and Sherbourne, 1992), consisting of 5 items with a 5-point Likert-type scale (1 = Never – 5 = All the time). An example item is: “In the last month, I have felt discouraged and sad.” The total score is calculated by adding up the direct scores and transforming the total scores into a scale ranging from 0 to 100. A higher score indicates better mental health. Cronbach’s alpha for T1 was 0.74 and for T2 was.79.

Control variables. Analyses were controlled by gender (1 = men; 0 = woman), age (measured in years) and number of economically dependent family members. The choice of these variables was based on previous research findings indicating the pandemic context affected the mental health of women and the elderly to a greater extent (Robinson et al., 2022). In addition, the application of FWAs also presented differences by gender, age, and number of children (Russell et al., 2009; Bouziri et al., 2020; Kotey and Wark, 2020), which have shown that participation in FWAs has a gender and age focus.

### Analytical procedure

Descriptive, correlational and reliability analyses were performed using SPSS 24 software. Prior to the estimation of the path analysis, confirmatory factor analysis (CFA) was performed for the measures of work overload and mental health. Model fit was evaluated using the chi-squared test (*χ*2), goodness of fit (GFI), comparative fit index (CFI), Tucker–Lewis index (TLI), root mean square error of approximation (RMSEA), and standardized root mean square residual (SRMR). According to Kline (2005), an acceptable model fit for CFI, TLI and GFI of >0.90 and for RMSEA and SRMR <0.08 were set.

Hypotheses were tested using two autoregressive models. First, the mediation effect of work overload (Time 2) in the relationship between WSF (Time 1) and mental health (Time 2) controlling the effects of work overload and mental health in Time 1 were tested. Second, the first stage moderation model (Edwards and Lambert, 2007) where the moderating effect applies to the first stage of the indirect effect of WSF (Time 1) on mental health (Time 2) was tested. Then, a moderator was added (WFH at Time 1), and its interaction with WSF (Time 1) in the relationship between WSF and work overload was tested. In this instance, the moderated mediation model was tested using the bootstrapping procedure (Hayes, 2013), which generates standard errors and 95% bias-corrected confidence intervals (Cis) around the indirect effects. To compare the mediation and moderated mediation models, the chi-square difference statistic was used. CFAs and path analyses were conducted using AMOS version 24.

## Results

Before testing the research hypotheses, CFA for work overload and mental health measures were conducted using maximum likelihood. The CFA of work overload at Time 1 and Time 2 showed adequate fit: *χ*^2^_(8)_ = 17.725; *χ*^2^/df = 2.215; GFI = 0.973; CFI = 0.978; TLI = 0.958; RMSEA = 0.076; SRMR = 0.030. The CFA of mental health at Time 1 and Time 2 also showed an adequate fit: *χ*^2^_(32)_ = 57.389; *χ*^2^/df = 1.793; GFI = 0.945; CFI = 0.954; TLI = 0.932; RMSEA = 0.066; SRMR = 0.059. In both cases, the results of factorial invariance across time, based on a comparison of an unconstrained model to a model with the loadings for corresponding items at T1 and T2 constrained to be equal, indicate that models were not significantly different (work overload: Δχ^2^_(2)_ = 5.79, *p* = 0.055; mental health: Δ*χ*^2^_(4)_ = 7.69, *p* = 0.103), supporting factorial invariance.

The means, standard deviations, reliabilities, and correlations among key variables are shown in Table 1. Given that not all control variables show significant relations with our study variables, only gender has been included in analyses testing the hypotheses.

**Table 1:** Descriptive statistics and correlation matrix among the study variables.

		Mean	S.D.	1	2	3	4	5	6	7	8	
1	Gender	1.54	0.50									
2	Age	42.96	13.28	−0.03								
3	Dependants	1.13	1.48	−0.06	0.09							
4	WFH T1	0.26	0.44	0.04	−0.07	−0.08						
5	WSF T1	0.46	0.50	−0.08	0.03	0.06	0.20^**^					
6	Work overload T1	2.84	1.32	−0.05	−0.08	0.08	0.02	−0.08	(0.79)			
7	Work overload T2	2.93	1.28	0.06	−0.13	−0.07	−0.03	−0.16^*^	0.40^**^	(0.80)		
8	Mental Health T1	77.92	15.72	−0.21^**^	0.07	0.12	−0.05	0.14^*^	−0.35^**^	−0.14^*^	(0.74)	
9	Mental Health T2	76.15	15.67	−0.30^**^	0.12	0.11	0.01	0.10	−0.16^*^	−0.28^**^	0.48^**^	(0.79)

Cronbach’s α is shown on the diagonal between brackets. ^*^p < 0.05; ^**^p < 0.01 (one-tailed tests). WFH, working from home. WSF, work schedule flexibility.

To test hypotheses 1, 2, and 3 the mediation model showed a satisfactory fit to the data [*χ*^2^_(129)_ = 210.658; *χ*^2^/df = 1.633; GFI = 0.902; CFI = 0.924; TLI = 0.910; RMSEA = 0.055; SRMR = 0.063]. The results of the path analysis are shown in Table 2.

**Table 2:** Summary of structural equations in the path analysis.

Equation	Mediation model			Moderated mediation model		
	*β*	*SE*	*R^2^*	*β*	*SE*	*R^2^*
1. DV: work overload (T2)			0.22^***^			0.29^***^
Work overload T1	0.45^***^	0.08		0.47^***^	0.08	
Gender	0.04	0.12		0.06	0.12	
WSF (T1)	−0.14^*^	0.13		−0.23^**^	0.15	
WFH (T1)				−0.23^*^	0.22	
WSF X WFH (T1)				0.29^*^	0.30	
2. DV: Mental health (T2)			0.40^***^			0.42^***^
Mental health (T1)	0.51^***^	0.09		0.51^***^	0.09	
Gender	−0.20^**^	0.11		−0.20^**^	0.11	
WSF (T1)	0.01	0.11		−0.01	0.12	
WSF X WFH (T1)				0.03	0.17	
Work overload (T2)	−0.25^**^	0.07		−0.26^**^	0.07	

DV, dependent variable. Regression coefficients are standardized. ^*^p < 0.05; ^**^p < 0.01, ^***^p < 0.001. WFH, working from home. WSF, work schedule flexibility.

Hypothesis 1 postulated that WSF would be negatively related to work overload. The result obtained, after controlling variables and work overload at T1, indicates that WSF (T1) was in fact negatively related to work overload (T2; *β* = −0.14; *p* < 0.05). Therefore, this result supports hypothesis 1.

Hypothesis 2 stated that work overload would be negatively related to mental health. In this case, the results show that, after controlling mental health at T1, work overload at T2 was in fact negatively related to mental health at T2 (*β* = −0.25; *p* < 0.01). Therefore, this result supports hypothesis 2.

Hypothesis 3 stated that work overload (T2) would mediate the relationship between WSF (T1) and mental health (T2). In this case, the total effect (*β* = 0.04, *SE* = 0.07, 95% CI: −0.08 to 0.14) and direct effect (*β* = 0.01, *SE* = 0.07, 95% CI: −0.12 to 0.01) of WSF (T1) on mental health (T2) were not significant. However, the relationship between WSF (T1) and mental health (T2) through work overload (T2) was confirmed as expected and is significant (indirect effect: *β* = 0.06, *SE* = 0.02, 95% CI: 0.01 to.15). These results reveal an inconsistent mediation due to the fact that a mediated effect has a different sign than direct effect (MacKinnon et al., 2000). Therefore, work overload (T2) mediates the relationship between WSF (T1) and mental health (T2). Thus, hypothesis 3 is supported.

To test hypotheses 4 and 5, the moderator (WFH at Time 1) and its interaction with WSF at Time 1 were included. This moderated mediation model showed a satisfactory fit to the data [*χ*^2^_(160)_ = 250.915; *χ*^2^/df = 1.568; GFI = 0.923; CFI = 0.931; TLI = 0.919; RMSEA = 0.052; SRMR = 0.061]. Also, this model presents a better fit compared to the mediation model [Δ*χ*^2^_(31)_ = 40.257, *p* < 0.01].

**Figure 2:**
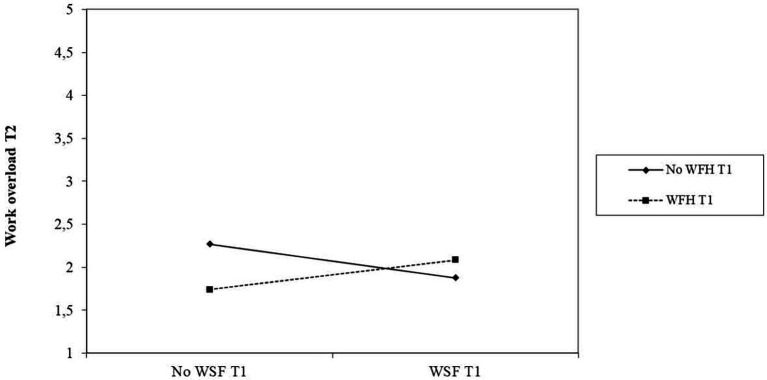
Teleworking (T1) as moderator of the relationship between WSF (T1) and work overload (T2). WFH, working from home. WSF, work schedule flexibility.

Hypothesis 4 stated that WFH (T1) would moderate the relationship between WSF (T1) and work overload (T2). The results obtained indicate that WFH T1 was significantly related to work overload at T2 (*β* = −0.23; *p* < 0.05). Also, the interaction between WSF and WFH at T1 was significantly related to work overload at T2 (*β* = 0.29; *p* < 0.05). To clarify these results, the interaction was graphically illustrated, plotting the moderator variable (WFH T1) using the two values of the moderator variable (0 = No WFH; 1 = WFH) as seen in Figure 2. In this case, the slope representing those who were not WFH was significant (*t* = −2.054; *p* < 0.05), but not for those who were WFH (*t* = 1.010; *p =* 0.314). Thus, this result supports hypothesis 4.

Finally, hypothesis 5 postulated that the positive indirect effect of WSF on mental health through work overload would be moderated by WFH, where the indirect effect would be stronger when workers were not WFH. The significance of the indirect effects was tested by means of maximum likelihood bootstrapped standard error (see MacKinnon, 2008), using the percentile bootstrap method, which maintains a good balance between Type I error rates and power (Hayes and Scharkow, 2013). The bias-corrected percentile bootstrap method further revealed a significant moderated mediation effect, *β* = −0.16, *SE* = 0.09, 95% CI = [−0.35 to −0.04], in which WFH moderated the mediating effect of WSF on mental health through work overload overtime. The conditional indirect effect of WSF (T1) on mental health (T2) *via* work overload (T2) was statistically significant for the respondents who were not WFH at T1, *β* = 0.12, *SE* = 0.06, 95% CI = [0.04 to 0.24]. In contrast, this indirect effect was non-significant for respondents who were WFH at T1, *β* = −0.02, *SE* = 0.05, 95% CI = [−0.12 to 0.05]. This result partially supports hypothesis 5.

Regarding control variables, only gender (*β* = −0.20; *p* < 0.01) was significantly related to mental health (T2). In this case, women presented higher levels of mental health in comparison with men.

## Discussion

According to the observed results, the aim of this study was achieved. Regarding the proposed hypotheses, there are several results to discuss.

First, regarding the effects of FWAs on work overload, a negative relation between WSF and work overload over time (H1) was observed. Although this relationship has been scarcely studied it is theoretically consistent to think that WSF increases employee control and autonomy, which in turn helps to cope with work overload, there are not many studies reporting this relationship during or before the pandemic. In spite of this, our results are in line with Van Steenbergen et al. (2018), who found that flexible schedule arrangements lead to decreasing mental demands and work overload.

Second, a negative relationship was observed between work overload and mental health (H2), which is consistent with previous studies such as Bowling et al. (2015), Melchior et al. (2007), Shultz et al. (2010), and Su et al. (2018). Also, a longitudinal study by de Beer et al. (2016) found that work overload indirectly negatively affects mental health *via* burnout. Other than in the healthcare sector, there is no published research on this relationship conducted during the pandemic context in organizational settings. This result therefore contributes to a better understanding of the effects of work overload on employee mental health in other settings.

Third, although our results showed that both types of FWA separately have a negative relationship with work overload over time, we found teleworking during COVID-19 pandemic acted as a moderator on the relationship between WSF and work overload (H4). Specifically, the negative effect of WSF on work overload was not significant for those who were also WFH. In other words, the lower work overload experienced by workers with WSF, was not observed in workers who experienced WSF and teleworking. Then, combining WSF and WFH can be counterproductive to reduce work overload because WFH in the absence of fixed working hours may imply for the worker a greater sense of availability and surveillance (Wang et al., 2020), which exacerbates the negative aspects of teleworking, such as the blurring of the boundaries of the daily working day, leading to long working hours and even night and weekend work (Eurofound, 2017). This may also be due to the fact that teleworking was mandatory during the pandemic and was therefore implemented without adequate preparation in response to the crisis. Recent studies found evidence supporting this claim. For example, a study conducted by Escudero-Castillo et al. (2021) during the pandemic, found that teleworkers have experienced less self-perceived well-being than non-teleworkers. Also, Jamal et al. (2021) reported that teleworking during the pandemic can be understood as a demand because it includes work extensification and intensification (Brammer and Clark, 2020). Moreover, Shiri et al. (2022), in their systematic review, found that FWAs generate long working hours which could be detrimental to mental health. Therefore, the moderated effect of teleworking observed in this study is in line with other research and shows how teleworking can dampen the positive effects of WSF when implemented without proper preparation, potentially diminishing the benefits that WSF can have on work overload over time.

Fourth, the evidence obtained supports the mediation model (H3) and the moderated mediation model proposed (H5) where WSF had a positive impact on employee mental health over time through a decrease in work overload. However, this mediation mechanism occurred when employees were not teleworking. For those who were teleworking during the pandemic, the relationship between WSF and work overload over time was not significant and, therefore, WSF did not impact teleworker mental health over time through decreasing work overload. Previous studies have shown similar results regarding the differences between teleworkers and non-teleworkers. Hayman (2010) compared the effects of WSF and teleworking on work overload and stress and his findings suggest that only WSF decreases work overload and increases work/life balance. In addition, Mendonça et al. (2022) reported that teleworking during the pandemic was associated with other demands such as imagined surveillance and communication overload, which negatively impacted mental health and quality of life.

### Implications for research and practice

This study has several theoretical implications. First, results provide evidence regarding the JD-R model (Demerouti et al., 2001) in terms of the consequences of FWAs over time during the pandemic. These results suggest, in this context, that WSF acts as a resource supporting coping with work demands, thus reducing work overload over time, indirectly affecting employee mental health. This reinforces the JD-R model with respect to the control that workers have over carrying out their tasks. In simpler terms, when workers have control over when to perform their work, the discomfort generated by work overload decreases. However, WFH, due to its mandatory application during the pandemic, was found to engender a series of consequences beyond the pre-pandemic applications. In this case, workers did not have control over deciding where to perform their work. Instead, it was imposed, indicating that in this context it cannot be considered a resource but a demand. This is in line with what Van Steenbergen et al. (2018) established that FWAs can decrease mental demands and work overload, but when mandatory, they have a negative impact on employee autonomy.

Secondly, the relationship between different FWAs also has implications for conservation of resources (COR) theory (Hobfoll, 1989) as far as how various flexibility-related arrangements interact in the pandemic context. In this vein, as WSF offers employees control over when they perform their work, it could allow them to better manage their resources on both personal and work-related levels, in line with the COR theory regarding ‘resources caravans’ (Hobfoll, 2011). However, although teleworking can grant employees control over where they can work, a mandatory form of implementation does not allow for better management of their resources. According to the COR theory, this could lead to a spiral loss of resources (Hobfoll et al., 2018), such as when more resources must be invested to respond to simultaneous role demands in the form of caring for others or attending meetings, which leads to diminished recovery time. Therefore, the combination of both forms of flexibility in the pandemic made teleworking counteract the resource generation involved in WSF.

Thirdly, results obtained contribute to the body of knowledge of FWAs. The longitudinal design used in the study allows more light to be shed on the consequences of FWAs on worker health over time, and establishing causal relationships among the study variables. Although there is still no evidence identifying which combination of FWAs provides the greatest benefits to employee health (Shifrin and Michel, 2021), the complex model proposed here advances our understanding of the effects of combining FWAs has on employee mental health.

Some practical implications can be derived from this study. First, although the pandemic precipitated the implementation of FWAs, in its aftermath, it is probable that the future of work will continue to move toward a FWAs modality. Scientific research such as this can provide organizations with an awareness of the positive and negative consequences of FWAs, in order to make informed decisions about how to approach their implementation. In addition, employers can use these results to inform their development of appropriate interventions to address any negative consequences of FWAs. Secondly, regarding the teleworking modality, these results illustrate the importance of establishing an informed plan before implementation to protect employee mental health. As International Labour Organization (2020b) recommend it could be useful to reach a mutual agreement with employees on the application of these modalities, offer degrees of autonomy and control over their work, recognize that offline time is needed, and provide employees with the physical, technological, and educational resources necessary for the proper performance of their work. For organizations that promote WSF and WFH at the same time, it would be advisable to put margins to flexibility by limiting the number of working hours to allow workers due rest or not implement one autonomous FWA together with a mandatory FWA. Finally, it is valuable for organizations to consider the importance of employee perception of control in the performance of their tasks. These results suggest that the impact of flexible working hours on coping with work reduces work overload over time. Also, it will be essential to consider the teleworkability of the role in terms of whether its different tasks are compatible with a teleworking modality (Eurofound, 2020).

### Possible limitations and directions for future research

The present study has some limitations. Firstly, although a longitudinal design with two measurement points was implemented, it’s relevant to note that our outcome and the mediator variable were measured simultaneously, which may have inflated the observed relationship between them. Although the effect of measurement point in T1 was controlled, it will be important for future researchers to consider different measurement points to test this mediation model. Secondly, all the data used was obtained through self-reporting measures, which could affect results due to common-method variance (Podsakoff et al., 2003). However, the correlations among the study variables differ in size, and the Harman single factor test showed an explanatory factor of 23.45%. Therefore, common-method variance does not significantly affect the relationship of the study variables. Thirdly, there are variables that have not been contemplated in the design which may explain the mechanisms of the relationship between studied variables. For example, both control and autonomy could play important roles in the relationship between FWAs and work overload. Teleworking entails several aspects that affect work overload, such as time pressure (Wöhrmann and Ebner, 2021), technostress (Rohwer et al., 2022), telepressure, and workplace monitoring (De’ et al., 2020). Moreover, burnout and work–family conflict (Lapierre et al., 2015; Barriga Medina et al., 2021) could also influence the relationships established in the model. Therefore, future studies should consider the use of these variables to gain a deeper understanding of the relationships established in the model. Also, dichotomous variables reduce the variability of FWAs measures, therefore for future studies the use of continuous variables is recommended. Fourthly, it is important to consider the context of the pandemic when interpreting the results, since COVID-19 engendered uncertainty, fear, confinement, etc., negatively impacting mental health of the population (Robinson et al., 2022). Therefore, in future, it is advisable to replicate the study during a context without a health emergency, to examine the implementation of FWAs in a voluntary setting.

## Conclusion

There are two main conclusions regarding the application of FWAs by organizations to cope with the consequences of the pandemic on the labor market. First, WSF generated positive effects on mental health over time through a decreased work overload, but only in those employees who were not also working from home. And second, the use of mixed FWAs, such as WSF and teleworking, did not generate additional benefits for employees. In fact, over time, teleworking mitigated the positive effects of WSF on work overload. Therefore, to maximize the positive effects of FWA implementation, organizations must be able to identify and balance job demands and resources to which employees are exposed. For example, when implementing teleworking, organizations can provide employees with resources such as high levels of control and autonomy and adjust demands such as high work overload to avoid negative consequences for employees.

In order to gain more insight into the impact of FWAs on employee mental health and work overload and understand the role of other variables underlying this relationship, future longitudinal research remains necessary.

## Data availability statement

The raw data supporting the conclusions of this article will be made available by the authors, without undue reservation.

## Ethics statement

The studies involving human participants were reviewed and approved by Ethics Committee of Adolfo Ibáñez University. The patients/participants provided their written informed consent to participate in this study.

## Author contributions

JY and MB contributed to conception and design of the study. JY organized the database and performed the statistical analysis. MB wrote the first draft of the manuscript. JY, MB, and CT-O wrote sections of the manuscript. All authors contributed to the article and approved the submitted version.

## Funding

The study was funded by the ANID-Millenium Science Initiative Program (NCS2021_033).

## Conflict of interest

The authors declare that the research was conducted in the absence of any commercial or financial relationships that could be construed as a potential conflict of interest.

## Publisher’s note

All claims expressed in this article are solely those of the authors and do not necessarily represent those of their affiliated organizations, or those of the publisher, the editors and the reviewers. Any product that may be evaluated in this article, or claim that may be made by its manufacturer, is not guaranteed or endorsed by the publisher.
